# Function-Oriented Graphene Quantum Dots Probe for Single Cell *in situ* Sorting of Active Microorganisms in Environmental Samples

**DOI:** 10.3389/fmicb.2021.659111

**Published:** 2021-05-25

**Authors:** Yeshen Luo, Fei Liu, Jianhua Song, Qian Luo, Yonggang Yang, Chengfang Mei, Meiying Xu, Bing Liao

**Affiliations:** ^1^Guangzhou Institute of Chemistry, Chinese Academy of Sciences, Guangzhou, China; ^2^State Key Laboratory of Applied Microbiology Southern China, Guangdong Institute of Microbiology, Guangdong Academy of Sciences, Guangdong, China; ^3^University of Chinese Academy of Sciences, Beijing, China

**Keywords:** graphene quantum dots, bioimaging, functional bacteria, single-cell *in situ* sorting, environmental samples

## Abstract

Functional microorganisms play a vital role in removing environmental pollutants because of their diverse metabolic capability. Herein, a function-oriented fluorescence resonance energy transfer (FRET)-based graphene quantum dots (GQDs-M) probe was developed for the specific identification and accurate sorting of azo-degrading functional bacteria in the original location of environmental samples for large-scale culturing. First, nitrogen-doped GQDs (GQDs-N) were synthesized using a bottom-up strategy. Then, a GQDs-M probe was synthesized based on bonding FRET-based GQDs-N to an azo dye, methyl red, and the quenched fluorescence was recovered upon cleavage of the azo bond. Bioimaging confirmed the specific recognition capability of GQDs-M upon incubation with the target bacteria or environmental samples. It is suggested that the estimation of environmental functional microbial populations based on bioimaging will be a new method for rapid preliminary assessment of environmental pollution levels. In combination with a visual single-cell sorter, the target bacteria in the environmental samples could be intuitively screened at the single-cell level in 17 bacterial strains, including the positive control *Shewanella decolorationis* S12, and were isolated from environmental samples. All of these showed an azo degradation function, indicating the high accuracy of the single-cell sorting strategy using the GQDs-M. Furthermore, among the bacteria isolated, two strains of *Bacillus pacificus* and *Bacillus wiedmannii* showed double and triple degradation efficiency for methyl red compared to the positive control (strain S12). This strategy will have good application prospects for finding new species or high-activity species of specific functional bacteria.

## Introduction

Identification, isolation, and domestication of particular functional microorganisms from the environment are necessary for the construction of bioresource reservoirs and environmental bioremediation (Berry et al., 2015). Classical methods that are widely used to detect and sort functional bacteria are time-consuming and take days or even weeks for screening or enriching target strains in selective media. To increase the detection efficiency, some new methods have been developed in recent years, including Raman-stable isotope probing (Song et al., 2017), immunomagnetic separation (Wen et al., 2018), droplet microfluidic technologies (Watterson et al., 2020), and fluorescence *in situ* hybridization in combination with fluorescence-activated cell sorting (Grieb et al., 2020). However, these methods do not allow the detection and screening of target bacteria from complex environmental samples based on their degradation functions. Moreover, the protocols of these methods employing these chemical or polynucleotide probes often result in reduced cell activity or even cell death, which renders the isolation and breeding of functional microorganisms futile (Batani et al., 2019).

In a previous study reported by our research group, a non-toxic silica nanoparticle probe in combination with fluorescence-activated cell sorting was used to detect and screen bacteria with degrading functions (Luo et al., 2020). To improve the screening efficiency for identifying these bacteria from the environment, graphene quantum dot (GQD) probes have been used as suitable alternatives to silica nanoparticles. GQD probes show excellent biocompatibility, and they easily penetrate the cell membrane and enter the cell owing to their small size. They constitute single or few layers of graphene particles that exhibit tunable surface functionality and long-term resistance to photobleaching (Castillo-Henriquez et al., 2020; Alavi et al., 2021). GQDs based on an “off-on” FRET mechanism have been widely used in the detection of various microorganisms. For example, Dehghani et al. (2020) designed a new fluorescence immunosensor based on GQDs for the detection of *Campylobacter jejuni*. Therefore, GQDs are an efficient tool for the detection and screening of functional bacteria.

Herein, we synthesized a function-oriented probe graphene quantum dots (GQDs-M) based on FRET which showed high sensitivity and selectivity for azo-respiration functional bacteria. This GQDs-M also showed high feasibility in visualizing azo-respiration functional bacteria in pure-cultured and environmental samples. Moreover, it is proposed that the estimation of environmental functional microbial populations based on bioimaging is a new method for rapid preliminary assessment of environmental pollution levels. Upon combining the single-cell sorting platform with the function-oriented GQDs-M probe, azo-respiration functional bacteria can be accurately sorted from environmental samples based on fluorescence labeling, and do not require any pretreatment.

## Materials and Methods

### Materials, Strains, and Growth Conditions

All chemicals were analytical grade pure reagents purchased from Aladdin^®^ or Macklin^®^ and used without further purification or treatment. Ultrapure water was produced by an ultrapure water instrument (18.2 MΩ cm @ 25°C, Synergy). Phosphate buffer saline (PBS) solution (NaCl 8 g/L, KCl 0.2 g/L, Na_2_HPO_4_ 1.42 g/L, KH_2_PO_4_ 0.27 g/L, and pH 7.0) was prepared using ultrapure water. LM medium (Lactate medium, Yeast extract 0.5 g/L, Na_2_HPO_4_⋅12 H_2_O 17.09 g/L, KH_2_PO_4_ 3 g/L, NaCl 0.5 g/L, NH_4_Cl 1 g/L, L-lactic acid sodium salt 5 g/L, and pH = 7.2) was prepared using ultrapure water. Luria-Bertani (LB) medium (Terptone 10 g/L, yeast extract 5 g/L, and NaCl 10 g/L) was prepared using ultrapure water.

The microorganisms *Shewanella decolorationis* S12, *Escherichia coli* S17-1, *Lysinibacillus* sp. GY32, *Bacillus terrae* RA9, *Bosea thiooxidans* BI-42, *Lysinibacillus pakistanensis* NCCP-54, and *Nocardia coeliaca* DSM 44595 were isolated from the environment and maintained in our laboratory. Environmental samples were sampled from several rivers in Guangdong, China (Baihua Chong, N23°09′26.10″ and E113°41′22.96″; #31–Wu Chong, N23°05′41.54″ and E113°27′10.46″; #15–Shen Chong, N23°06′20.21″ and E113°24′42.89″; #1–Ma Chong, N23°37′65.72″ and E113°21′31.61″; #14–Bei Jiang tributary, N23°20′49.05″ and E112°95′26.71″; #6–Liuxi Chong, N23°20′49.05″ and E112°95′26.71″; RG–Rong Gui, N22°78′09.69″ and E113°25′12.33″; and ST–Shengtong, N23°28′03.59″ and E113°07′75.55″).

The microbes were individually cultured in 4 mL of LB medium overnight at 30°C to the stationary phase. One hundred μL of each culture were transferred to fresh LB medium and incubated overnight at 30°C to the logarithmic phase. Cells were then (i) harvested at 7,500 rpm for 10 min, (ii) washed three times with PBS, and (iii) diluted in LM medium to OD600 ∼0.1 to remove the remaining LB media, which would generate a background fluorescence signal during bioimaging.

### Preparation of GQDs-N

Nitrogen-doped GQDs (GQDs-N) were prepared using a modified bottom-up strategy with a new precursor (Wang et al., 2014). Perylene (0.25 g, 99%) was subjected to nitrification in concentrated HNO_3_ (20 mL) at 80°C under reflux and stirring for 12 h. After nitrification, the mixture was diluted with ultrapure water (250 mL) and filtered through a 0.22-μm microporous membrane to remove the acid, and the orange intermediate product 3, 4, 9, and 10-tetranitroperylene was isolated (Eberson and Radner, 1985; Gade et al., 2002). The orange product was further dispersed in an ammonium hydroxide solution in ultrapure water (30 mL, 1.2 M) by ultrasonication (100 W, 40 kHz) for 2 h. The suspension was transferred to a poly(tetrafluoroethylene)-lined autoclave (50 mL) and heated at 200°C for 10 h. After cooling to room temperature, the product containing water-soluble GQDs was filtered through a 0.22-μm microporous membrane to remove the insoluble carbon product and further dialyzed in a dialysis bag (retained molecular weight: 3,500 Da) for 1 week to remove sodium salt and unfused small molecules. The purified black GQDs-N were subjected to vacuum freezing and drying for structural analysis and property determination.

### Preparation of GQDs-M

Graphene quantum dots were prepared according to a method reported in the literature (Liu et al., 2015) with a slight modification, *i.e*., 0.1 mmol methyl red was dissolved in H_2_O/DMSO solution (1:2) and 90 mg of 1-(3-Dimethylaminopropyl)-3-ethylcarbodiimide hydrochloride (0.47 mmol) were added, followed by the slow addition of 0.01 M HCl to adjust the pH of the reaction solution to 5. After stirring for 30 min at room temperature, *N*-hydroxy succinimide (50 mg, 0.4 mmol) was added to the mixture, and the GQDs-N (50 mg) in water solution (10 mL) were then added dropwise to the mixture, followed by pH adjustment of the mixture to 9.0, using 0.01 M NaOH. The reaction solution was gently stirred for 48 h in the dark. Finally, the resulting solution was dialyzed against ultrapure water for 3 days. The purified GQDs-M were subjected to vacuum freezing and drying for structural analysis.

### Bioimaging and Detection of Functional Bacteria

The bacteria were cultured with GQDs-M (10 μg/mL) in LM to initiate decolorization. The culture was then sampled after 12 h, centrifuged, washed, and resuspended in sterile phosphate-buffered saline. All cultures were diluted to an OD_600_ of 0.1, to monitor under laser scanning confocal microscopy (LSCM). The environmental samples were added. *Lysinibacillus* sp. GY32 (long linear bacteria) was used as the positive control, followed by the addition of GQDs-M (10 μg/mL). The environmental samples were also cultured for 12 h before LSCM monitoring.

### Single Cell Sorting of Environmental Samples

Ten environmental samples from Guangdong Province, including five water samples, Ma Chong (#1), Liuxi Chong (#6), Bei Jiang tributary (#14), Shen Chong (#15), and Wu Chong (#31), were used, along with five sediment samples: Rong Gui (RG) aerated for 1 month (RG Ca), RG sediment microbial fuel cells over 1 month of operation (SMFC1 and SMFC2), and Shentong (ST). Each environmental sample was divided into two groups, and the pure strain S12 was used as a positive control against the environmental samples. A total of 21 sets of samples were analyzed. The probe, GQDs-M, was added to the environmental samples, followed by overnight anaerobic culture. The sorting experiment was successively performed using an LSCM and a single-cell precision sorter (PRECI SCS and HOOKE Instruments). The prepared bacterial liquid was sampled on the sorting chip of a single-cell sorter. The fluorescence signal was first observed under LSCM, and the fluorescence of the bacteria was estimated. The selected bacteria were then ejected using a laser beam controlled by PRECI SCS software (Gan et al., 2020). After the bacteria were sorted, they were transferred to LB medium for large-scale culturing. Expanded cultures were sequenced to identify the selected species. Next, fluorescence verification and azo decolorization function verification of the selected species were performed.

### Characterization

The thickness of the GQDs was determined using atomic force microscopy (BioScope Resolve, Bruker). Transmission electron microscopy (TEM) observations were performed using an H-7650, Hitachi. High-resolution transmission electron microscopy (HR-TEM) observations were performed using a field-emission transmission electron microscope (JEM-2100F, JEOL). The chemical states of the GQDs were analyzed by a multifunctional photoelectron spectrometer (XPS, Axis Ultra DLD, Kratos). Spectral data acquisition and processing were carried out using XPSpeak 41 software, and detailed analyses of C 1s and N 1s core levels were performed. Absorption and fluorescence spectra were recorded at room temperature on an ultraviolet and visible spectrophotometer (UV-2600, SHIMADZU) and fluorometer (LS-45, PerkinElmer), respectively. Absolute quantum yield and fluorescence lifetime were recorded using a fluorometer FLS980 (with an integration sphere, Edinburgh Instruments). Fourier transform infrared (FTIR) spectra of the dried samples were recorded using a FTIR spectrometer (TENSOR II, Bruker). Raman spectra were recorded on a Raman spectrometer (LabRAM Araims, HORIBA Jobin Yvon). Laser scanning confocal microscopy (LSCM, LSM 700 Zeiss) was used to monitor the distribution of fluorescence in the bacteria.

## Results and Discussion

### Preparation and Characterization of GQDs

**SCHEME 1:**
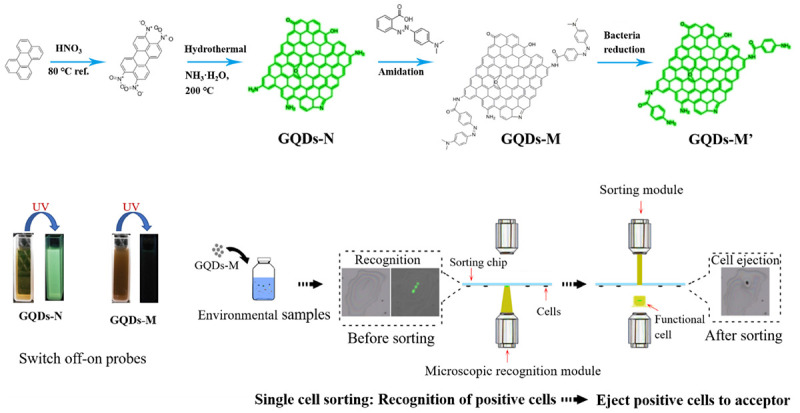
Strategy for using graphene quantum dot (GQD) probes for single-cell sorting.

Nitrogen-doped GQDs were obtained using a bottom-up strategy (Scheme 1). Perylene was subjected to nitration using hot HNO_3_ to afford the precursor, 3, 4, 9, and 10-tetranitroperylene (1H-NMR and 13C-NMR in Supplementary Figures 1, 2) (Nordbotten et al., 1984; Cho, 1995). The precursor has four positively charged sites of NO_2_ groups, which allow nucleophilic substitution reactions to occur with several alkaline species such as NH_3_ and NH_2_NH_2_ that are added to the hydrothermal media, implying that the precursor can be easily fused with graphitized carbon materials at a low temperature (200°C) (Wang et al., 2014). Therefore, ammonium hydroxide was selected to synthesize GQDs-N. In addition, the introduction of these alkaline species allowed the formation of water-soluble OH- and amine-functionalized GQDs. X-ray photoelectron spectroscopy (XPS) data shows that the GQDs-N (Supplementary Figure 3a) contain 3.51% N (Supplementary Table 1). As shown in Supplementary Figure 4, the FTIR spectra of GQDs-N shows a strong and broad vibration peak at ∼3,400 cm^–1^, corresponding to the O–H bond, and a peak at ∼3,200 cm^–1^, attributed to the N–H bond, confirming that GQDs-N contains hydroxyl and amino groups.

**FIGURE 1:**
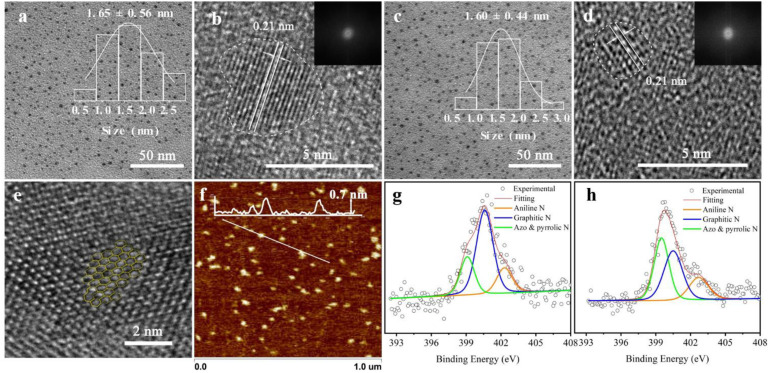
Micromorphology and properties of graphene quantum dots (GQDs). **(a,b)** Transmission electron microscopy (TEM) and high-resolution (HR)-TEM images of GQDs-N [inset: particle size distribution in the TEM image and fast Fourier transform (FFT) pattern in the HR-TEM image]. **(c,d)** TEM and HR-TEM data of GQDs-M (inset: particle size distribution in the TEM image and FFT pattern in the HR-TEM image). **(e)** Part of the honeycomb structure of the GQDs. **(f)** Atomic force microscopy height sensor image of GQDs-N (inset: height profile along the white line). High-resolution N 1s XPS data of GQDs-N in panel **(g)** and GQDs-M in panel **(h)**; the concentrations of the corresponding functional groups are included in Supplementary Table 2.

Herein, the preparation of the probes, GQDs-M, is carried out through covalent bonding of GQDs-N with a commercial azo dye, methyl red, *via* amidation. The FTIR spectrum confirms the presence of three characteristic peaks for amide bonds in the GQDs-M after modification. As shown in Supplementary Figure 4, the spectrum of GQDs-M shows several characteristic peaks in comparison to the GQDs-N, which are listed as follows: ν_–C__=__O_ (amide I) at ∼1,634 cm^–1^, δ_N__–__H_ (amide II) at ∼1,570 cm^–1^, ν_–C__–__N_ (amide III) at ∼1,382 cm^–1^, and –N=N– peak at 1,442 cm^–1^ (Guerrero et al., 2014). Also, the UV spectrum of GQDs-M show an additional absorption peak at 520 nm after modification, which differs from the spectral characteristics of GQDs-N (Supplementary Figure 5). The XPS data of GQDs-M also shows that the ratio of N changes to 6.64 (Supplementary Figure 3b and Supplementary Table 1). The high-resolution N 1s XPS spectra (Figures 1g,h and Supplementary Table 2) reveal three types of N-related bonds, namely, aniline N (∼399.2 eV), graphitic N (∼401.6 eV), and azo and pyrrolic N (∼402.7 eV) (Gassman et al., 1992; Dai et al., 1993; Tang et al., 2014). After modification by methyl red, the azo and pyrrolic N contents of the GQDs increase. These results indicate that methyl red is covalently bonded to the GQDs-N in GQDs-M.

The structure of the functionalized GQDs were further characterized using TEM, HR-TEM, and atomic force microscopy. The atomic force microscopy image shows that the thickness of the GQDs is 0.4–0.7 nm (Figure 1f), corresponding to single-layer graphene. The TEM images of the GQDs show that all the functionalized GQDs are uniform and monodisperse (Figures 1a,c). The average lateral sizes of GQDs-N (inset in Figure 1a) and GQDs-M (inset in Figure 1c) are ∼1.65 ± 0.56 and ∼1.60 ± 0.44, respectively. After GQDs-N were modified to become GQDs-M, their dimensions did not change significantly. Upon further observation using HR-TEM (Figures 1b,d) and analysis of the FFT patterns (insets in corresponding images) of a single particle of the functionalized GQDs, a clear graphene lattice with a spacing of 0.21 nm corresponds to that of the graphene (100) plane. However, analysis of the FFT patterns (insets in the corresponding images) indicates that the functionalized GQDs have poor crystallinity. Moreover, a high-magnification HR-TEM image of a single GQD shows a typical graphene honeycomb structure (Figure 1e). Owing to the introduction of N and O, the honeycomb structure becomes a distorted structure with five-, seven-, and ten-membered rings (Toh et al., 2020). The Raman *I*_D_/*I*_G_ ratios (where *I*_D_ and *I*_G_ are the D- and G-band Raman intensities, respectively) are widely used to evaluate the quality of the carbon material (Pachfule et al., 2016). Therefore, Raman analysis was performed to confirm this result. The Raman spectrum of these sheets shows the characteristic spectral bands of graphene, D band (∼1,338 cm^–1^), and G band (∼1,580 cm^–1^), accompanied by a defect in the 2D band (∼2,830 cm^–1^). As shown in Supplementary Figure 6, the *I*_D_/*I*_G_ ratios for GQDs-N and GQDs-M are 0.99 and 0.97, respectively. These values are significantly larger than those of reported GQDs (0.83–0.87) (Wang et al., 2014). The results indicate that these have more *sp*^3^ carbon defects, and the GQD structures are similar to those of graphene oxide nanosheets. Therefore, functionalized GQDs exhibit excellent fluorescence properties because the fluorescence emission of GQDs is believed to originate from the recombination of electron-hole pairs localized within small *sp*^2^ carbon clusters embedded within the *sp*^3^ matrix (Ding et al., 2018).

### Optical Properties of Functionalized GQDs

**FIGURE 2:**
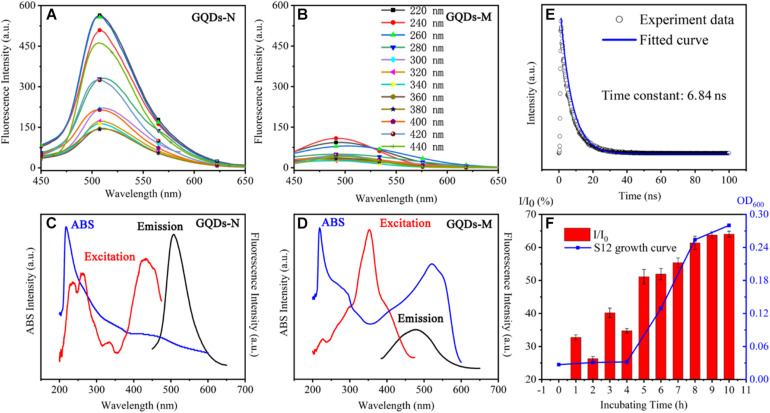
Optical properties of functionalized GQDs. Fluorescent spectra of GQDs-N in panel **(A)**, GQDs-M in panel **(B)**. UV-visible absorption, emission, and excitation spectra of GQDs-N in panel **(C)**, and GQDs-M in panel **(D)**. Time-resolved fluorescence spectrum of GQDs-N in panel **(E)**. Changes in the fluorescence intensity *I*/*I*_0_ at 520 nm as a function of time of GQDs-M1 incubation with strain S12 (1–10 h). *I* represents the fluorescence intensity of GQDs-M and *I*_0_ indicates the fluorescence intensity of GQDs-N in panel **(F)**.

The functionalized GQDs exhibited excellent optical performance. As shown in Scheme 1, GQDs-N showed green fluorescence upon UV irradiation (365 nm), but the fluorescence was quenched after GQDs-M were linked with methyl red because of the FRET process (Liu et al., 2015). GQDs-N showed a high absolute quantum yield of 18% in deionized water. The absorption spectrum (Figure 2C) of GQDs-N exhibits a pronounced excitonic absorption band centered at ∼220 nm and a weak absorption band at ∼450 nm. The emission peak at 520 nm can be excited over a wide wavelength range, with bands centered at approximately 220, 260, and 470 nm (emission/excitation spectrum in Figure 2C). The main excitation bands are in agreement with the corresponding excitonic absorption bands, suggesting that the emission is characterized by band-edge exciton-state decay. These intrinsic fluorescence characteristics are also exhibited in the fluorescence decay curve (Figure 2E), which shows mono-exponential decay and a long lifetime (6.84 ns) (Wang et al., 2014). GQDs-M exhibits different optical properties. Its absorption spectrum (Figure 2D) exhibits three pronounced excitonic absorption bands centered at approximately 220, 290, and 520 nm, with an optical absorption edge at ∼600 nm. The absorption range of GQDs-M is consistent with that of GQDs-N connected to methyl red (highlighted in Supplementary Figure 5). The fluorescence intensity of GQDs-M is very low, and it can only be excited in a narrow wavelength range (emission/excitation spectrum in Figure 2D). The FRET process is a nonradiative process whereby an excited state donor transfers energy to a proximal ground state acceptor through long-range dipole-dipole interactions (Sapsford et al., 2006). The acceptor may fluoresce after absorbing the energy of the fluorescence emitted by the donor, but it may also be quenched. In this study, the acceptor was quenched. At the same excitation wavelength, the fluorescence intensity of GQDs-M was only ∼10% of that of GQDs-N (Figures 2A–D). The efficiency E_FRET_ of the FRET quenching can be determined from steady-state measurements as given in Eq. 1 (Hameed et al., 2021).

(1) $$E=1-\frac{F_{D\,A}}{F_{D}},$$

where *F* is the relative donor fluorescence intensity in the absence (*F*_*D*_) and presence (*F*_*DA*_) of the quencher. From the available data, and using Eq. 1, we calculated the FRET efficiency *E*_FRET_ for GQDs-M to be 0.93. Therefore, no fluorescence was observed upon UV irradiation of the GQDs-M.

In addition, GQDs-N showed good chemical stability and photostability. The emission and excitation intensities of GQDs (Supplementary Figures 7a,b) decrease under strongly acidic conditions (pH < 4), but do not change under neutral or alkaline conditions. The photostability of the GQDs was tested by 2 h of continuous radiation using a xenon lamp; the fluorescence intensity of the GQDs did not change during the experiment (Supplementary Figure 7c).

Previous studies have shown that the fluorescence of the probe recovers when the azo moiety is degraded by microorganisms (Luo et al., 2020). Therefore, GQDs-M can serve as an off-on switch probe for monitoring azo-respiration functional bacteria. To evaluate this capability, the fluorescence intensity recovery ratio *I*/*I*_0_ was determined, where *I* corresponds to the fluorescence intensity of GQDs-M, which was incubated with the azo-respiration functional bacterial strain *S. decolorationis* S12, and *I*_0_ corresponds to the fluorescence intensity of GQDs-N (the precursor of GQDs-M). As shown in Figure 2F, *I*/*I*_0_ is positively correlated with the growth curve of strain S12. The *I*/*I*_0_ ratio increases with an increase in the incubation time; after 8 h, S12 grows to the logarithmic stage and the *I*/*I*_0_ value reaches 61%. With the growth of S12, GQDs-M acts as an electron acceptor and the azo bond is reduced by azo respiration, such that GQDs-M again emits fluorescence. This indicates that GQDs-M can act as an off-on switch probe for monitoring azo-respiration functional bacteria. This also indicates the good biocompatibility and non-toxicity of GQDs-M to S12.

### Bioimaging and Functional Microorganism Proportion Estimation

**FIGURE 3:**
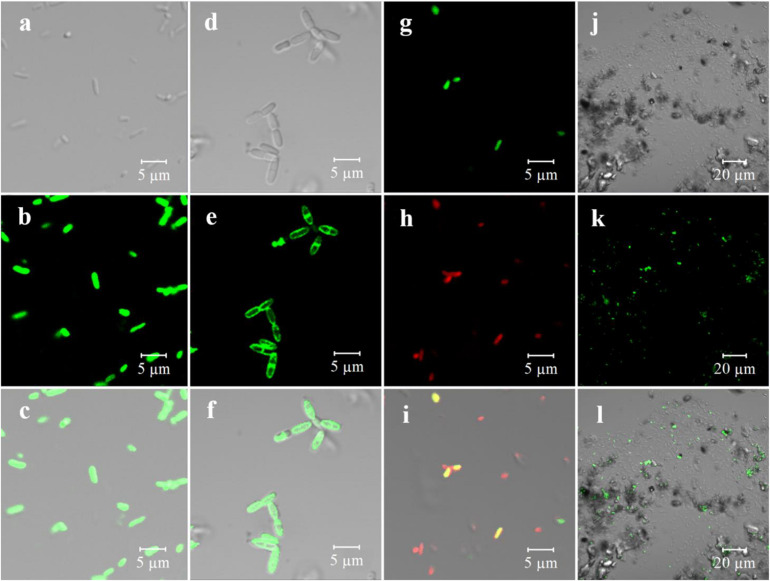
Bioimaging of pure culture of microorganism and real samples with GQDs-M (1 mg/mL) including fluorescent channel, bright-field, and merged channel. **(a)** S12-bright field; **(b)** S12-GQDs-M channel; **(C)** S12-merged channel; **(d)** BI-42-bright field; **(e)** BI-42-GQDs-M channel; **(f)** BI-42-merged channel; **(g)** artificial microbial community (S12, *Escherichia coli*, C1)-GQDs-M channel; **(h)** artificial microbial community-DAPI channel; **(i)** artificial microbial community-merged channel; **(j)** environmental sample (Baihua Chong)-bright field; **(k)** environmental sample-GQDs-M channel; and **(l)** environmental sample-merged channel.

Owing to the specificity of the off-on optical property, GQDs-M was expected to serve as an excellent fluorescent probe for the bioimaging and monitoring of azo-respiration functional bacteria. To evaluate this capability, several strains, including long lines, slender rods, and other different forms of bacteria, were selected for experimental verification. *E. coli* S17-1 could not perform azo-respiration, and *S. decolorationis* S12, *Lysinibacillus* sp. GY32, *B. terrae* RA9, *B. thiooxidans* BI-42, *L. pakistanensis* NCCP-54, and *N. coeliaca* DSM 44595 with azo-degradation function (Luo et al., 2020) were employed. As shown in Supplementary Figure 8, all strains except *E. coli* can completely degrade methyl red at a concentration of 0.1 M within 8 h. All strains were grown in a culture medium containing GQDs-M (10 μg/mL), and confocal microscopic images were obtained using a 488-nm laser at a low voltage. Strain S12 with the degradation function and *E. coli* without the degradation function were used as models. As shown in Supplementary Figure 9, with an increase in the incubation time, an increasing number of S12 bacteria show fluorescence. When the culture time was greater than 6 h, fluorescence was detected in the complete S12 flora. However, even after 21 h of incubation, *E. coli* did not fluoresce. These results indicate that GQDs-M can be used as a probe to determine if the microorganisms exhibit an azo degradation function. In addition, confocal detection of other azo-respiration functional bacterial strains revealed that they all showed fluorescence (Supplementary Figure 10). This indicates that the GQDs-M is not specific to azo-respiration functional bacteria, but has universality to bacteria capable of azo-reduction. Furthermore, the magnified images in Figures 3a–f show that S12 and BI-42 exhibit fluorescence with different localization, *i.e*., for BI-42, it is localized on the cell membrane and for S12, whole-body fluorescence is observed. This indicates that GQDs-M may be used to localized the azo-reducing process. Azo bond is electron deficient and readily accepts electrons from redox proteins or mediators during anaerobic respiration of microorganism and is reduce (Cao et al., 2013; Liu et al., 2017). Previous studies have confirmed that S12 shows both extracellular and intracellular azo degradation pathways, causing S12 to fluoresce whole-body (Chen et al., 2010). For BI-42, an azo-reducing process may mainly occur on the cell membrane.

To further verify the specificity of the probe, an artificial community including S12 and non-degradable bacteria, *E. coli* and *Sphingobium hydrophobicum* C1 (slender rod and coccus), was constructed. As shown in Figures 3g–i, the green channel corresponds to the GQDs-M fluorescence signal, and the red channel is correlated to the commercial dye DAPI, which is mainly used to determine the presence of cells. In this community, only S12 shows green fluorescence, and *E. coli* and C1 are non-fluorescent. This indicates that the probe cannot only be used for pure cultures, but also for analysis of the microorganism community.

Graphene quantum dots were further applied to environmental samples to detect azo-respiration functional bacteria. As river sediments are rich in biomass, the sediments from three polluted rivers in Guangzhou, China (Baihua Chong, Wu Chong, and Shen Chong) were sampled. One gram of sediment was diluted with 50 mL of LM. Following the addition of GQDs-M into the culture, strain GY32 was added to the mixed culture as a positive control because its long linear morphology could be clearly identified. After culturing for 12 h, the samples were monitored using CLSM. As shown in Supplementary Figure 10, all three river samples contained large amounts of microbes, including the long linear strain GY32, which is a fluorescent positive control. Simultaneously, the bacteria in the original environment can be distinguished as bacteria showing fluorescence and those without fluorescence. In other words, azo-respiration functional bacteria were detected in all three rivers. Additionally, as shown in Figures 3j–l, no false-positive fluorescent interference was observed, even in the presence of sand, algae, or suspended impurities in the sample. Therefore, the proportion of azo-respiration functional bacteria in the environment was estimated using the ratio of bacteria with and without fluorescence. Herein, ten groups of environmental samples were evaluated, as shown in Supplementary Figure 11. The proportion of azo-respiration functional bacteria in water samples is typically lower than that in sediments, and the proportion in the polluted river Wu Chong (#31) with the highest turbidity is higher than in any other river water sediment. In addition, the proportion of bacteria in the sediments from the same location changed after various treatments. After aeration for 1 month, the proportion of azo-respiration functional bacteria in the sediment of RG decreased more than in the original sediments, to a value that was half of the proportion for the original sediment. The proportion of azo-respiration functional bacteria in RG sediment microbial fuel cells over 1 month of operation also decreased. These results indicate that the microbial community is affected by environmental substrates and environmental pollution levels (Kong et al., 2019). Because of aeration and SMFC operation, the environmental samples are repaired to a certain extent (Anawar and Chowdhury, 2020; Khan et al., 2021). Specialization of the community occurs through the emergence and selection of the most adapted phylotypes, which can be dye-tolerant or decolorizing species (Yu et al., 2015). In polluted environmental samples, bacteria with respiratory versatility are significantly enriched (Yang et al., 2018). Therefore, the estimation of environmental functional microbial populations based on bioimaging will be a new method for rapid preliminary assessment of environmental pollution levels.

### Single Cell Sorting

**FIGURE 4:**
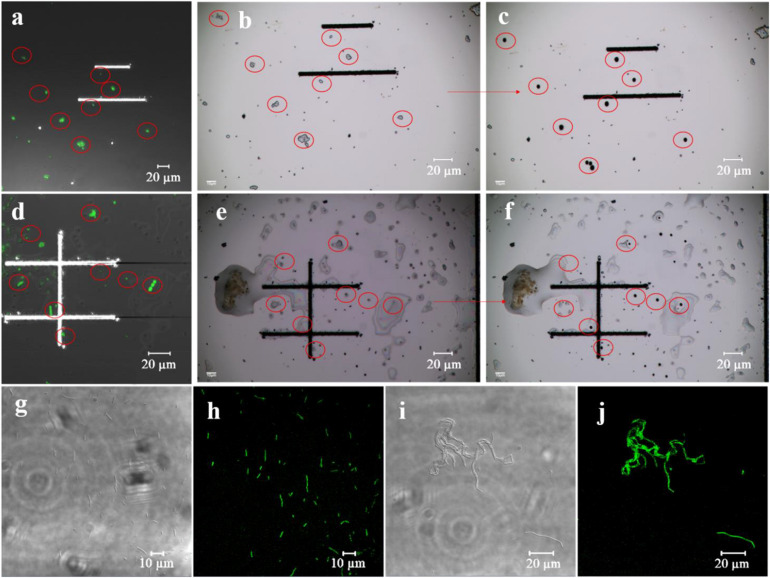
Single-cell sorting of environmental samples. **(a–f)** Single-cell sorting; **(a)** (#31) and **(d)** (RG) are fluorescent images of the sorting chip, **(b,e)** indicate sorting chip images before sorting, and **(c,f)** represent sorting chip images after sorting. **(g–j)** Fluorescence verification; **(g,h)** are bright field and fluorescent images of sorted strain S1A1, and **(i,j)** correspond to the sorted strain RCA.

Many azo-respiration functional bacteria are present in the environmental samples based on estimation using the GQDs-M probe. Their precise isolation from the environmental samples and cultivation is important. Therefore, the fluorescent probe was combined with a single-cell sorting technique (PRECI SCS) to screen for azo-respiration functional bacteria. The environmental samples were dropped on a sorting chip (a glass slide designed for single cell ejection) and placed in a semi-dry state, so the relative position of the bacteria remained unchanged (Figure 4). First, the fluorescence signal was observed to mark the positive bacteria (Figures 4a,d), and then the corresponding positive bacteria were sorted on the visual single cell sorter (Figures 4b–c,e–f). The sorted bacteria were cultured in LB medium for large-scale culture. Among the 21 groups of samples, 14 groups of sorted bacteria could be cultured in the most basic LB medium, accounting for 66.7% of the samples. Supplementary Table 3 summarizes the sorting results for all the samples. Seventeen strains were isolated from the 21 sample groups. The extracted DNA of the obtained strains was amplified by 16S PCR and then subjected to 16S rRNA gene sequencing. The results are shown in Supplementary Table 3; strains 1A, 1GG, STG, 31A, 31GG, and S2A are *Bacillus pacificus*; strains RCA, RCGA, and RA1 are *Bacillus velezensis*; strains S1A1 and S1A2 are *Stenotrophomonas pavanii*; the positive control is S12A for *S. decolorationis* S12; strain S1GA1 is *Rhizobium skierniewicense*; strain STA is *Bacillus wiedmannii*; strain 15A is *Ochrobactrum haematophilum*; strain RA2 is *Bacillus albus*; and strain S1A3 is *Alcaligenes faecalis*.

**FIGURE 5:**
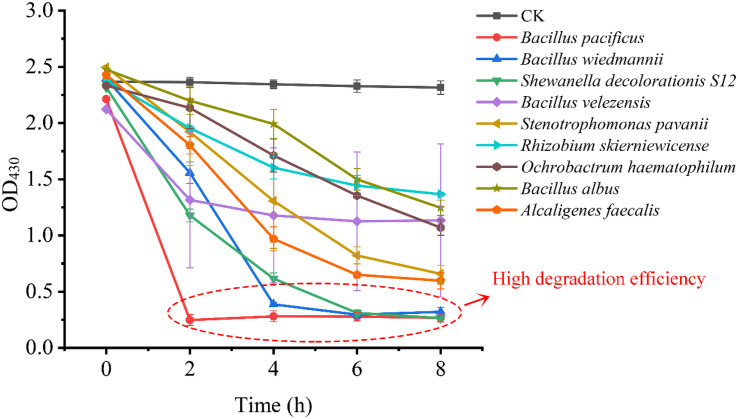
Verification of azo decolorization function of sorted bacteria.

The main aim of using GQDs-M is to achieve species-independent and function-oriented single-cell sorting; therefore, in addition to ensuring the taxonomic coincidence of sorted cells, it is necessary to reconfirm their ability to degrade pollutants. First, fluorescence verification of the sorted bacteria was performed. As depicted in Figures 4g–j and S12, the bioimaging results suggest that all strains show a fluorescent signal when cultured with GQDs-M. Moreover, the morphologies of the bacteria observed using a confocal microscope were consistent with the results of 16S rRNA gene sequencing, *i.e*., the cell morphologies of the same bacteria were identical. For example, RCA, RCGA, and RA1 correspond to the same bacteria, *B. velezensis*, and were observed using a confocal microscope as long line bacteria. In addition, their decolorization ability was verified. As shown in Figure 5, all strains show some decolorization ability. Two strains (*B. pacificus* and *B. wiedmannii*) show double and triple degradation efficiency for methyl red compared to that of strain S12. Under identical conditions, 0.1 mM methyl red is completely degraded within 2 h by *B. pacificus* and within 4 h by *B. wiedmannii*, which is faster than the 6 h required by S12. Other bacteria showed lower efficiency than S12. In summary, all sorted bacteria showed an azo degradation function, indicating that GQDs-M is a function-oriented probe, and the single-cell sorting strategy combined with GQDs-M with a visual single-cell sorter is sensitive and accurate to 100%. The above results suggest that the azo-degradation function-oriented GQD probe is specific and accurate. The specificity of GQDs-M is mainly dependent on the FRET mechanism. In this study, FRET probes, GQDs-M, were constructed using GQDs and methyl red. GQDs-M specifically target bacteria that can degrade the azo dye, but theoretically can be generalized to target microorganisms with specific respiratory metabolic functions if a corresponding FRET mechanism is suitably matched.

## Conclusion

In summary, GQDs-N were synthesized *via* a bottom-up approach using perylene. GQDs-N showed excellent optical properties, such as high quantum yield, long-term optical stability, and acid-base stability. The surfaces of GQDs-N were modified to form GQDs-M with an azo dye, affording optical properties constituting an off-on fluorescent switch, which can be used as a specific probe to accurately identify bacteria with azo respiratory activity. It is suggested that the estimation of environmental functional microbial populations based on bioimaging will be a new method for rapid preliminary assessment of environmental pollution levels. GQDs-M combined with a visual single-cell sorter affords a species-independent, culture-independent, and function-oriented method for the rapid, sensitive, and precise single-cell screening of azo respiratory microorganisms in environmental samples. This strategy can provide an alternative method to isolate various functional bacteria and new species or high-activity species of functional bacteria from the environment.

## Data Availability Statement

The raw data supporting the conclusions of this article will be made available by the authors, without undue reservation.

## Author Contributions

The manuscript was written through contributions of all authors. All authors have given approval to the final version of the manuscript.

## Conflict of Interest

The authors declare that the research was conducted in the absence of any commercial or financial relationships that could be construed as a potential conflict of interest.
