# Supplementary material for: Function-Oriented Graphene Quantum Dots Probe for Single Cell in situ Sorting of Active Microorganisms in Environmental Samples
Source: Front Microbiol. 2021 May 25;12:659111. doi: 10.3389/fmicb.2021.659111 (PMC8186282; doi:10.3389/fmicb.2021.659111)
Supplement: Supplementary file 1 [file Data_Sheet_1.docx]

**Function-oriented Graphene Quantum Dots Probe for Single Cell in Situ Sorting of Active Microorganisms in Environmental Samples**

Yeshen Luo,^1,2,3^ Fei Liu,*^2^ Jianhua Song,^2^ Qian Luo, ^2^ Yonggang Yang,^2^ Chengfang Mei,^2^ Meiying Xu,*^2^ Bing Liao,*^1^

^1^ Chinese Academy of Sciences, Guangzhou Institute of Chemistry, Guangzhou 510650, China

^2^ State Key Laboratory of Applied Microbiology Southern China, Guangdong Institute of Microbiology, Guangdong Academy of Sciences, Guangdong, China

^3^ University of Chinese Academy of Sciences, Beijing, 101408, China.


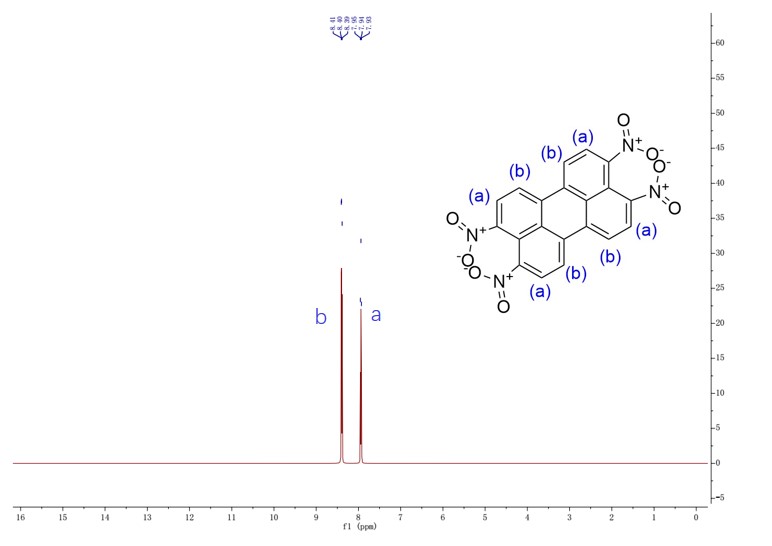


Fig. S1. The ^1^H-NMR spectra of 3, 4 9, 10-tetranitroperylene in DMSO.


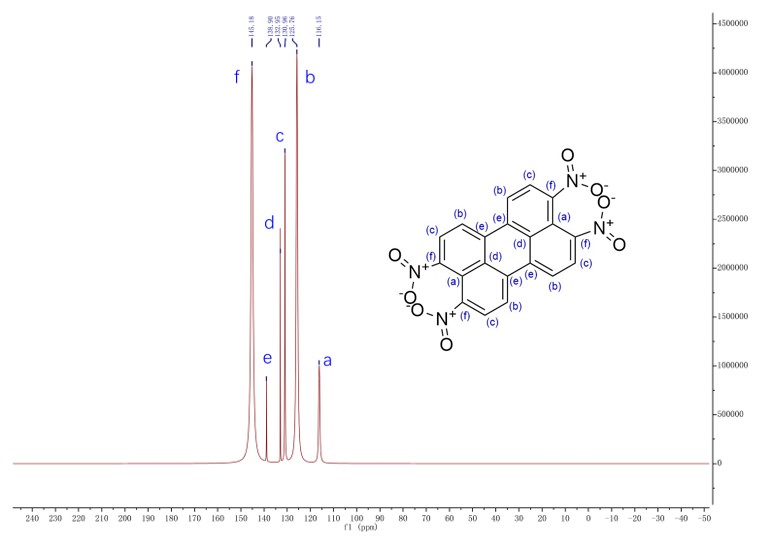


Fig. S2. The ^13^C-NMR spectra of 3, 4 9, 10-tetranitroperylene in DMSO.


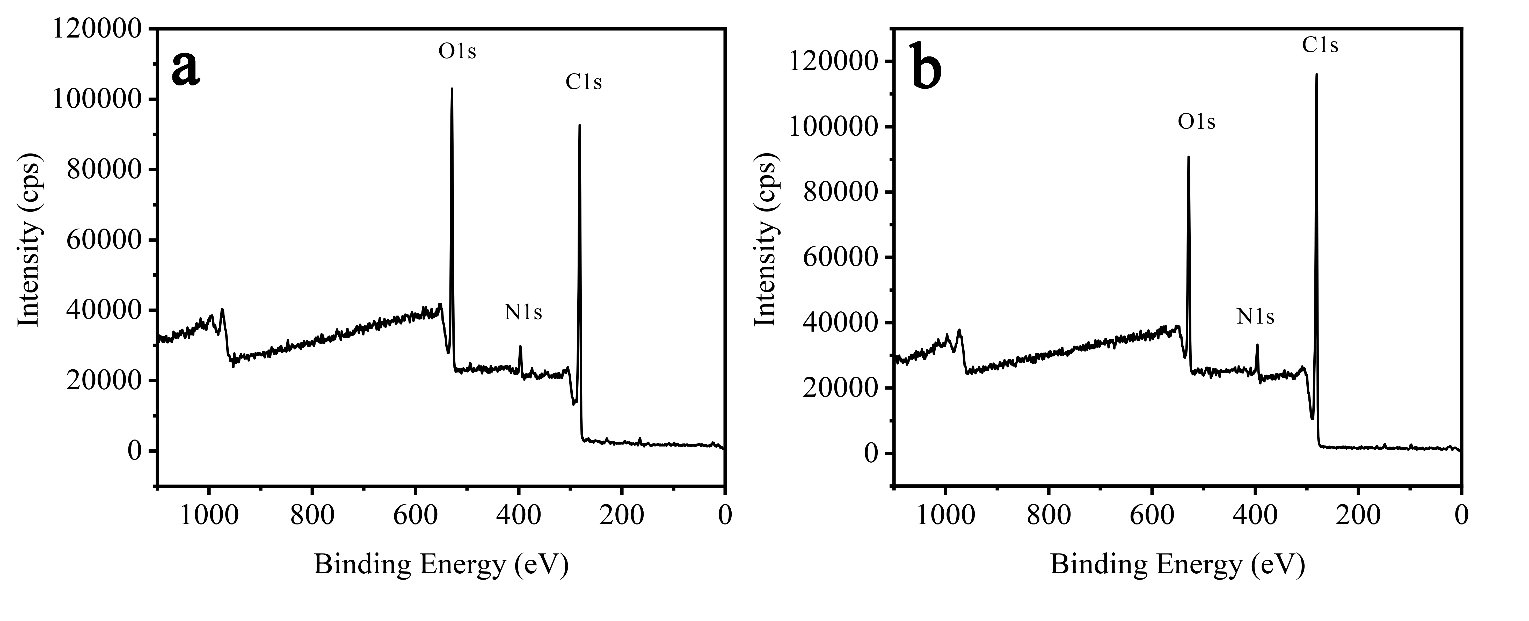
 Fig. S3. XPS data of GQDs-N (a) and GQDs-M (b). The concentration of the corresponding elements is displayed in Table S1.





Fig. S4. Fourier transform infrared spectra of GQDs.





Fig. S5. Absorption spectra of GQDs.





Fig. S6. Raman spectra of GQDs.


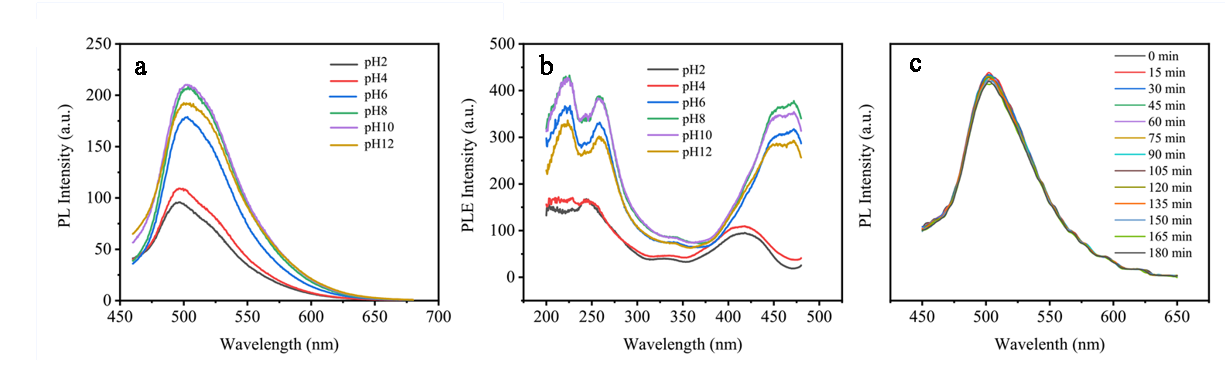


Fig. S7. Fluorescence emissions and excitation spectra of GQDs. a: Emission spectra of GQDs-N at different pH. b: Excitation spectra of GQDs-N at different pH. c: Emission spectra of GQDs-N under laser exposure time series.


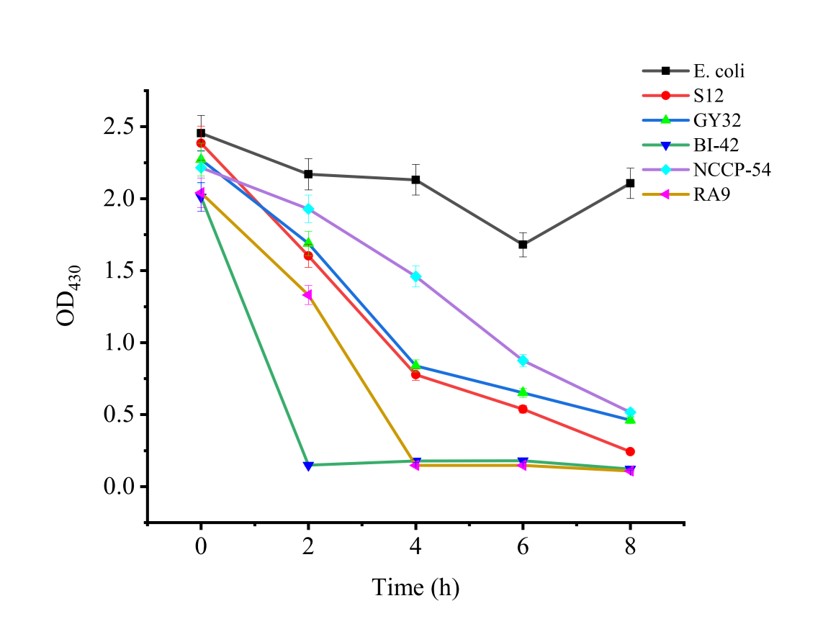


Fig. S8. Decolorization of methyl red by different microorganisms.


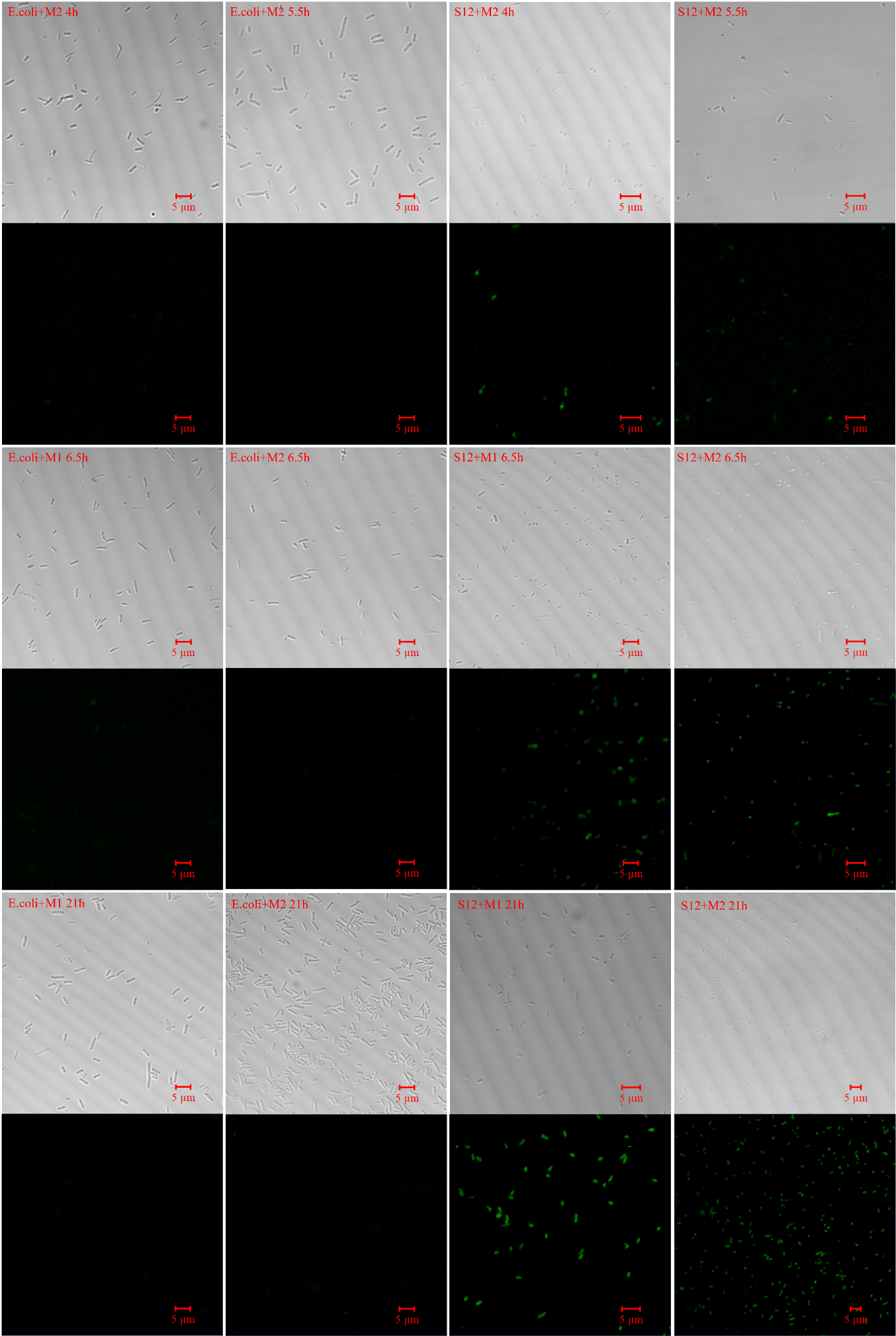


Fig. S9. Bioimaging of E. coli and S12 cultivated with GQDs-M in different time.


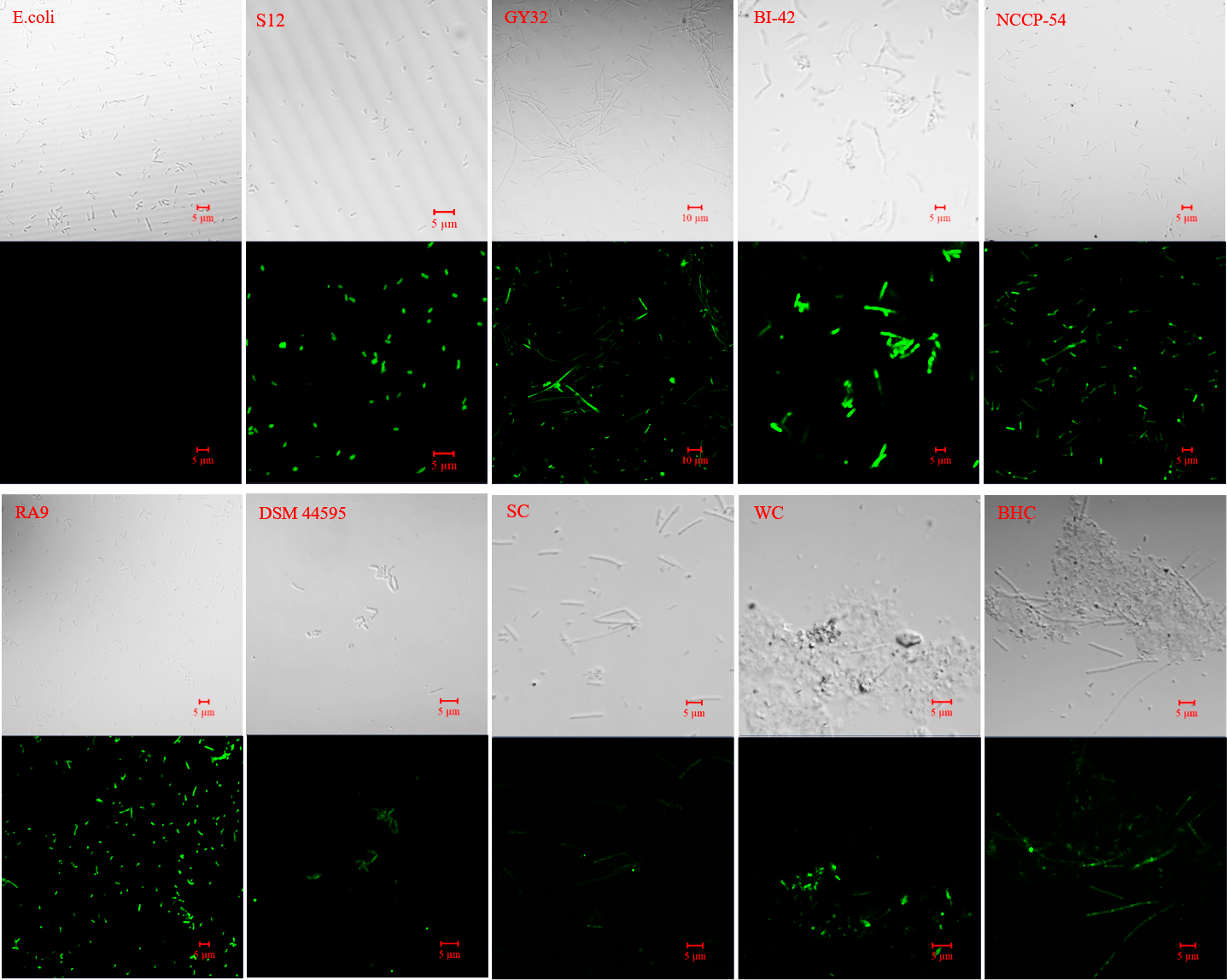


Fig. S10. Bioimaging of pure culture microorganism and real samples with GQDs-M (1mg/mL), GY32 (long linear bacteria) as a positive control added into the environmental samples.





Fig. S11. The azo-respiration bacteria ratio of environmental samples.


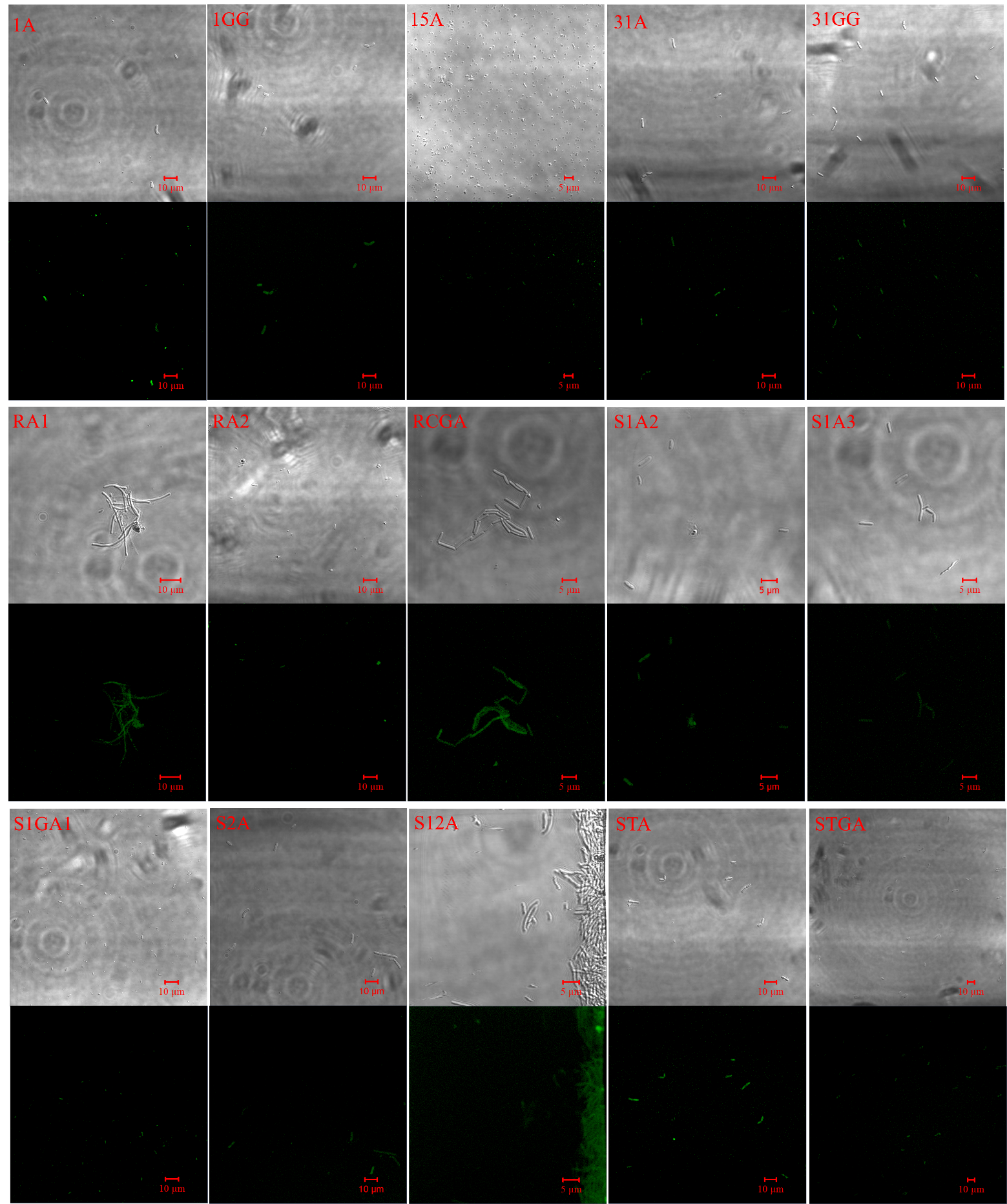


Fig. S12. Bioimages of sorted strains.

| Element | GQDs-N | GQDs-M |
| --- | --- | --- |
| C/Mass Conc (%) | 70.92 | 78.2 |
| N/Mass Conc (%) | 3.51 | 6.64 |
| O/Mass Conc (%) | 25.57 | 15.16 |

Table S1. The element content is calculated from XPS. The results are based on Fig. S3.

| Species of N | GQDs-N | GQDs-M |
| --- | --- | --- |
| Azo & pyrrolic N/Area ratio (%) | 23.0 | 40.3 |
| Graphitic N/Area ratio (%) | 60.0 | 40.5 |
| Aniline N/Area ratio (%) | 17.0 | 19.2 |

Table S2. The concentration of corresponding functional groups in the N 1s peak fitting of XPS. The results are based on Figs. 1g,h.

Table S3. Results and verification of in-situ sorting of environmental samples.

| Sample | Culture | Strain | name | Species identific | Fluorescence | Discoloration |
| --- | --- | --- | --- | --- | --- | --- |
| #1 | *√* | 1 | 1A | *Bacillus pacificus* | √ | √ |
| #1+GY32 | √ | 1 | 1GG | *Bacillus pacificus* | √ | √ |
| #6 | - | - | - | - | - | - |
| #6+GY32 | - | - | - | - | - | - |
| #14 | - | - | - | - | - | - |
| #14+GY32 | - | - | - | - | - | - |
| #15 | *√* | 1 | 15A | *Ochrobactrum haematophilum* | √ | √ |
| #15+GY32 | - | - | - | - | - | - |
| #31 | √ | 1 | 31A | *Bacillus pacificus* | √ | √ |
| #31+GY32 | √ | 1 | 31GG | *Bacillus pacificus* | √ | √ |
| ST | √ | 1 | STA | *Bacillus wiedmannii* | √ | √ |
| ST+GY32） | √ | 1 | STG | *Bacillus pacificus* | √ | √ |
| SMFC1 | √ | 3 | S1A1 | *Stenotrophomonas pavanii* | √ | √ |
|  |  |  | S1A2 | *Stenotrophomonas pavanii* | √ | √ |
|  |  |  | S1A3 | *Alcaligenes faecalis* | √ | √ |
| SMFC1+GY32 | √ | 1 | S1GA1 | *Rhizobium skierniewicense* | √ | √ |
| SMFC2 | √ | 1 | S2A | *Bacillus pacificus* | √ | √ |
| SMFC2+GY32 | - | - | - | - | - | - |
| RG | √ | 2 | RA1 | *Bacillus velezensis* | √ | √ |
|  |  |  | RA2 | *Bacillus albus* | √ | √ |
| RG+GY32 | - | - | - | - | - | - |
| RG Ca | √ | 1 | RCA | *Bacillus velezensis* | √ | √ |
| RG Ca+GY32 | √ | 1 | RCGA | *Bacillus velezensis* | √ | √ |
| S12 | √ | 1 | S12A | *Shewanella decolorationis* S12 | √ | √ |
